# Do Flavor Descriptions Influence Subjective Ratings of Flavored and Unflavored E-liquids Among Nonsmoking and Non-vaping UK Adolescents?

**DOI:** 10.1093/ntr/ntae054

**Published:** 2024-03-12

**Authors:** Maddy L Dyer, Steph F Suddell, Jasmine N Khouja, Michelle A Havill, Anna K M Blackwell, Olivia M Maynard, Marcus R Munafò, Angela S Attwood

**Affiliations:** School of Psychological Science, University of Bristol, Bristol, UK; Medical Research Council Integrative Epidemiology Unit at the University of Bristol, Bristol, UK; Trinity College Institute of Neuroscience (TCIN), Trinity College Dublin, Dublin, Ireland; School of Psychological Science, University of Bristol, Bristol, UK; Medical Research Council Integrative Epidemiology Unit at the University of Bristol, Bristol, UK; Department of Health and Social Care, Office for Health Improvement and Disparities, London, UK; Department of Psychology, University of Bath, Bath, UK; School of Psychological Science, University of Bristol, Bristol, UK; Medical Research Council Integrative Epidemiology Unit at the University of Bristol, Bristol, UK; School of Psychological Science, University of Bristol, Bristol, UK; Medical Research Council Integrative Epidemiology Unit at the University of Bristol, Bristol, UK; National Institute for Health Research Bristol Biomedical Research Centre, University Hospitals Bristol NHS Foundation Trust, Bristol, UK; School of Psychological Science, University of Bristol, Bristol, UK; Medical Research Council Integrative Epidemiology Unit at the University of Bristol, Bristol, UK

## Abstract

**Introduction:**

Youth use of electronic cigarettes (e-cigarettes) is rising globally and is associated with health harms. Flavor descriptions on e-liquid packaging may contribute to the appeal of e-cigarettes among youth. This study compared subjective ratings of e-liquid packaging flavor descriptions among nonsmoking and non-vaping UK adolescents.

**Aims and Methods:**

This was an online observational study in a UK sample of nonsmoking and non-vaping adolescents aged 11–17 years. The primary analyses compared flavored versus unflavored descriptions and the secondary analyses compared sweet flavor versus fruit flavor descriptions. Outcomes were packaging appraisal, packaging receptivity, perceived harm, and perceived audience.

**Results:**

The survey was completed by 120 participants (74% female). Packaging appraisal ratings were higher for e-liquids with flavored descriptions than unflavored descriptions (mean difference 5.9, 95% CI: 4.2 to 7.6, *p* < .001). Similarly, packaging receptivity ratings were higher for e-liquids with flavored descriptions than unflavored descriptions (mean difference 4.2, 95% CI: 2.8 to 5.6, *p* < .001). Participants also perceived e-liquids with flavored (vs. unflavored) descriptions as less “grown-up” (mean difference −5.2, 95% CI: −7.3 to −3.1, *p* < .001). However, ratings of perceived harm were similar for flavored and unflavored descriptions (mean difference −1.0, 95% CI: −2.6 to .5, *p* = .189).

**Conclusions:**

Although this study found differences in subjective ratings of e-liquids with flavored and unflavored descriptions, nonsmoking and non-vaping UK adolescents generally had low appraisal and receptivity for e-liquids and they perceived them as being “grown-up” and harmful.

**Implications:**

Youth use of electronic cigarettes (e-cigarettes) is increasing globally, leading to concerns about health harms. This study compared adolescents’ ratings of e-liquids with flavored versus unflavored descriptions and e-liquids with sweet flavor versus fruit flavor descriptions. This study adds to previous studies that have compared adolescents’ ratings of e-liquids with tobacco flavor versus non-tobacco flavor descriptions. Although packaging appraisal and receptivity ratings were higher (more positive) for e-liquids with flavored versus unflavored descriptions, overall, adolescents who do not smoke or vape had low appraisal and receptivity for e-liquids, and they perceived them as being “grown-up” and harmful.

## Introduction

Youth use of electronic cigarettes (e-cigarettes/vapes) is rising globally, which is a public health concern.^1,2^ In a survey of over 150 000 13–15-year-olds in 47 countries from 2015 to 2018, 9% reported using e-cigarettes (vaping) in the past 30 days.^3^ Selling e-cigarettes to anyone under 18 years of age and purchasing e-cigarettes for anyone under 18 years of age are criminal offenses in Great Britain.^4^ Yet in 2022, 16% of 11–17-year-olds had tried vaping (11% in 2021) and 7% were current users (3% in 2021).^5^ In 2023, the most frequently used e-cigarette product by 11–17-year-olds was a disposable vape (69%), a rise from 52% in 2022 and 8% in 2021.^5,6^ However, e-cigarette use among 11–17-year-olds who have never smoked cigarettes remains low with only 2% reporting at least monthly use.^5^

This increased prevalence is concerning because using nicotine-containing e-cigarettes is associated with mouth and throat irritation, headache, cough, and nausea,^7^ and nicotine is highly addictive when inhaled.^8^ Although the long-term health consequences of vaping are unknown,^2,9^ some suggest that youth vaping could impair lung function and act as a “gateway” to smoking cigarettes.^10–12^ Smoking is the biggest cause of preventable disease and death in the United Kingdom.^4^ However, it is unclear whether associations between youth vaping and adult smoking reflect a causal pathway; these findings may be explained by shared common causes of smoking and vaping.^13–15^

One factor that may be driving the increase in youth vaping is the flavors of e-cigarette products and how these are promoted, for example via descriptions on packaging. There are thousands of e-liquid flavors in some markets including fruit, sweet, dessert, menthol or mint, nut, spice, coffee, tea, and alcohol flavors, and many appeal to youth.^16,17^ The U.S. Food and Drug Administration and the World Health Organization argue that the way some e-cigarettes have been marketed to youth is specific and deliberate.^16,18^ Many nicotine-containing products include flavors that could appeal to youth, including sweet flavor descriptions such as bubble gum, marshmallow, and jelly babies. Two systematic reviews of cross-sectional and longitudinal studies predominantly from the United States and the United Kingdom found that the availability of flavors, particularly fruit and sweet flavors, decreased perceived product harm, increased willingness to try e-cigarettes, and increased initiation and continuation of e-cigarette use among youth.^19,20^ However, others suggest that “liking the flavors” (10%) is a less common reason for e-cigarette use among British youth who are never smokers compared to “just giving it a try” (65%).^5^

In response to the rise in youth vaping, several countries have restricted the sale of flavored e-cigarette products. Excluding countries that prohibit e-cigarette sales, three countries (Finland, Hungary, and Montenegro) have banned all flavors in e-cigarette products except tobacco flavor, and six countries (Denmark, Estonia, Germany, New Zealand, Philippines, and Saudi Arabia) have banned specific flavors.^16^ Flavored cartridge-based e-cigarettes are banned in the United States except for tobacco and menthol flavors.^21^ An independent review into smoke-free 2030 policies argued that the UK Government should prevent youth from vaping by banning “child-friendly” packaging and descriptions.^22^ When designing the current study, the UK had no e-cigarette flavor restrictions.^23^ However, in January 2024 the UK Government announced their intention to impose restrictions.^24^

Understanding how youth perceive flavored and unflavored e-cigarette products is important as perceptions can influence use. However, few studies have examined youth perceptions of unflavored e-liquids, distinguished between unflavored and tobacco flavors, and included nonsmoking and non-vaping youth specifically.^19^ Vasiljevic et al. found that nonsmoking and non-vaping English 11–16-year-olds rated adverts for flavored (vs. unflavored) e-cigarettes as more appealing, and they reported greater interest in buying and trying these products.^25^ In a United States study, e-cigarette users aged 11–16 years perceived flavored e-cigarettes as less harmful than non-flavored e-cigarettes.^26^ Another U.S. study found lower perceived harm was associated with greater interest in trying e-cigarettes among 13–17-year-olds, and perceived harm partially mediated the relationship between flavor and interest in trying e-cigarettes.^27^

Youth associate different e-cigarette flavors with different target audiences. For example, in one UK survey, 11–16-year-olds perceived a never-smoker of their age to be more likely than an adult smoker to try candy floss and cherry flavors.^28^ In another U.S. survey, most 14–21-year-olds thought that adverts for fruit- and dessert-flavored e-liquids targeted individuals about their age, and adverts for unflavored e-liquids targeted older individuals.^29^ Furthermore, 94% of participants thought the target age group for a cupcake-flavored e-liquid advert was younger than them.^29^

E-liquid flavors are important to adult vapers. In the United Kingdom, many smoking cessation experts caution that policy changes that restrict e-liquid flavors could have unintended consequences, for example, by reducing the appeal and effectiveness of e-cigarettes for smoking reduction and cessation.^30^ Therefore, the UK Government are carefully considering whether or how e-cigarette flavors and descriptions are restricted by legislation.^24,31^ In a UK qualitative study, some adult smokers reported that they would be more likely to continue smoking and some adult vapers reported that they would be more likely to relapse to smoking if only unflavored, tobacco-flavored, and menthol-flavored e-liquids remained on the UK market.^32^

## Current Study

This study compared subjective ratings of e-liquid packaging flavor descriptions among nonsmoking and non-vaping UK adolescents. The primary aim was to compare the effect of flavored versus unflavored descriptions on packaging appraisal and packaging receptivity for e-liquid products that were consistent across other packaging features (eg, colors, imagery, and branding). The secondary aim was to compare the effect of flavored versus unflavored descriptions on perceived harm of, and perceived audience for, the product. This study also compared the effect of sweet flavor versus fruit flavor descriptions on all four outcomes. Differences may exist because sweets have strong associations with youth and are consumed more by youth than by adults.^20,33^

We predicted higher (more positive) ratings of packaging appraisal and packaging receptivity for e-liquids with flavored (vs. unflavored) descriptions and sweet flavor (vs. fruit flavor) descriptions. We also predicted that e-liquids with flavored (vs. unflavored) and sweet flavor (vs. fruit flavor) descriptions would be perceived as more childish (less grown-up). While we predicted a difference in ratings, we did not have a strong directional hypothesis for the effect of e-liquid packaging flavor description on perceived harm.

## Materials and Methods

### Design

This was an online observational survey study. Subjective ratings of e-liquid packaging flavor descriptions (ie, the category of flavor description which appears on the e-liquid label/packaging) were examined using a within-participant design. Ratings of flavored versus unflavored descriptions and sweet flavor versus fruit flavor descriptions were compared. Outcome variables were packaging appraisal, packaging receptivity, perceived harm, and perceived audience. The study protocol was preregistered on the Open Science Framework (https://osf.io/zrxyw) and later updated during data collection due to a change in recruitment strategy (https://osf.io/39u6h). Ethics approval was obtained from the School of Psychological Science Research Ethics Committee at the University of Bristol (reference: 291020112404).

### Participants and Recruitment

Participants were recruited between March 2021 and December 2022. UK residents who were aged between 11 and 17 years, had normal or corrected-to-normal vision, and did not currently smoke or vape and had never regularly smoked or vaped, defined as not having tried smoking or vaping on more than 10 occasions, were included. The higher criterion of 100 occasions is commonly used across the life course for adults, but a lower level of exposure was necessary for our adolescent sample, and this criterion of 10 occasions has been used previously.^34^ Initially, participants were recruited via schools, academies, and colleges, hereafter referred to as “schools.” Teachers were contacted via email and social media. Schools that agreed to support participant recruitment were asked to send the study invitation letter and information sheet to parents or guardians of students aged 11–17 years. Three out of six schools agreed to this, two out of six schools granted permission to contact year 12–13 students only (16–17-year-olds), and one institution was a college admitting year 12–13 students only. Parents or guardians passed the survey link and password to their child(ren) if they consented to them participating. Participating schools received £100 regardless of the number of student responses.

Recruitment took place during the coronavirus disease-2019 (COVID-19) pandemic when schools and parents were facing considerable pressures, which hindered recruitment. Therefore, in June 2022, the study protocol was revised to allow 16–17-year-olds to participate directly and parental contact was only needed for potential participants aged 11–15 years. The study was advertised to parents and young people via charities, local authority public health teams, educational and youth organizations, Mumsnet, Gumtree, university newsletters, and social media.

### Materials and Measures

The data dictionary describes all measures and is available at the University of Bristol data repository (doi:10.5523/bris.qmqm15cey1ef27u3szoujxt5x).

#### Participant Characteristics

Participants provided their age, gender, and school name. School postcode was used as a proxy for socioeconomic status (ie, the degree to which an area had low to high progression to higher education) based on POLAR4 quintiles.^35^ Participants reported whether they had tried smoking or vaping, and whether their immediate family members and friends smoke or vape. Smoking susceptibility and vaping susceptibility were also measured. Total scores of three items each assessed participants’ perceived likelihood of smoking or vaping now, in the next year, or by age 18, ranging from 3 (low) to 12 (high).

#### E-liquid Packaging Flavor Descriptions

Using online searches, the study team identified five vaping brands that sold fruit-flavored, sweet-flavored, and unflavored e-liquids. This ensured that product images were matched across categories, for example in terms of design and branding. Because of the limited availability of unflavored e-liquids, the selected brands were not necessarily representative of the UK market, for example in terms of their packaging color, font, and imagery. However, this was considered the best approach because the effect of flavor descriptions was the primary focus of the study. E-liquids were purchased, matching for volume and nicotine concentration for the three flavor categories within brands. Different product sizes (10–50 mL) and nicotine concentrations (3–18 mg/mL) were selected across brands to show a range of products. Each product was photographed under standardized conditions. The stimuli set included 15 images (Table S1).

#### Subjective Ratings of Packaging

Rating scales were developed from a study evaluating adolescents’ ratings of cigarette packaging.^36^ For each image, 11 items were presented on scales ranging from 0 to 100. For each item, participants were asked “Using the sliders below, can you tell me the number that best describes the packaging?” The items included 10 used by Ford and colleagues: (1) “unattractive” to “attractive”, (2) “not eye-catching” to “eye-catching”, (3) “not cool” to “cool”, (4) “not at all harmful” to “very harmful”, (5) “boring” to “fun”, (6) “not meant for someone like me” to “meant for someone like me”, (7) “childish” to “grown-up”, (8) “puts me off smoking cigarettes” to “tempts me to smoke cigarettes”, (9) “I dislike this pack” to “I like this pack”, and (10) “I would not like to have this” to “I would like to have this”.^36^ One item (“not worth looking at” to “worth looking at”) was excluded from the original scale to reduce survey duration and due to its low relevance to the research question. An 11th item was added: (11) “puts me off vaping (using e-cigarettes)” to “tempts me to vape (use e-cigarettes)”. Ford and colleagues derived two primary composite measures: “packaging appraisal” (items 1, 2, 3, and 5) and “packaging receptivity” (items 6, 8, 9, 10—and the current study includes new item 11).^36^ Items 4 “perceived harm” and 7 “perceived audience” were analyzed separately.

### Procedure

The online survey was created using Qualtrics (www.qualtrics.com). Potential participants viewed a brief information sheet that included the study eligibility criteria and they provided checkbox assent to participate. Individuals who did not confirm that they met the eligibility criteria were not able to proceed to the next screen to start the study. Participants were informed that their answers would be kept private, and if they wanted to stop, they could close the survey and their answers would not be used. First, participants reported their age, gender, and school name. The task instructions were then presented with examples. Each image was shown with the 11 rating scales and there was no time limit for responding (Figure S1). Image presentation was randomized using the Qualtrics “Randomizer” and “Evenly Present Elements” functions so that each of the 15 images was shown once to each participant, but in no particular order. Rating scales were presented in a fixed order. Two attention check questions were included where participants were required to select a specified number on a rating scale. Participants answered the smoking and vaping questions and left comments in a free-text box. Finally, participants viewed the debrief webpage. If they entered the prize draw, they were redirected to another survey to preserve the anonymity of study data and they provided their contact details. There were five £50 vouchers and fifteen £20 vouchers available, and prize winners were selected at random in January 2023.

### Statistical Analyses

The target sample size of 205 participants was not reached, which would have enabled a comparison of three flavor description categories. However, the minimum target sample size of 120 participants was reached, which enabled a comparison of two flavor description categories (ie, flavored vs. unflavored). This analysis had 95% power at an alpha level of 0.005 to detect an effect size of Cohen’s *dz* = 0.4, and 80% power at an alpha level of 0.05 to detect a smaller effect size of Cohen’s *dz* = 0.3. Comparisons between sweet flavor and fruit flavor descriptions were secondary. A small effect size was chosen in the absence of a reasonable effect size from a similar published study. Using an effect size from a study examining subjective ratings of cigarette or alcohol packaging was considered unsuitable.

Analyses were conducted using Stata (SE V.15). Participant characteristics (age, gender, socioeconomic status, and smoking and vaping variables) were summarized using descriptive statistics. Paired sample *t*-tests were used to compare subjective ratings of e-liquid packaging flavor descriptions. Sensitivity analyses were conducted that excluded any participants who failed the attention checks. Finally, unplanned post hoc analyses explored participants’ perceptions of the “childish” to “grown-up” measure and their free-text comments.

## Results

Study data and analysis code are available at the University of Bristol data repository (doi:10.5523/bris.qmqm15cey1ef27u3szoujxt5x).

### Participant Characteristics

**Figure 1.**
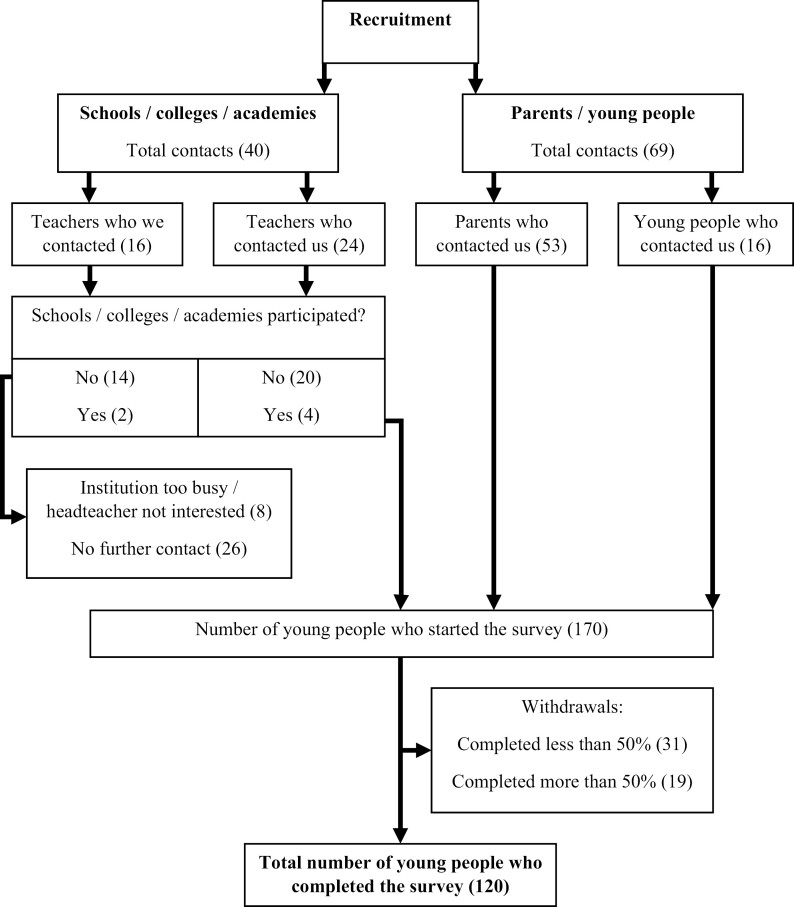
CONSORT diagram

The survey was started by 170 participants of whom 50 withdrew, leaving 120 participants (71%) in the final sample (Figure 1). Approximately 25% of participants were recruited before the recruitment strategy changed in June 2022. Participants (74% female) were aged between 11 and 17 years (*M* = 15.2, SD = 1.8; Table 1). Most participants had never tried smoking (83%) or vaping (77%), and had low smoking (*M* = 4.1, SD = 1.7) and vaping (*M* = 4.7, SD = 1.9) susceptibility. Most participants (74%) completed the survey in 30 minutes or less, which was the estimated response time. Four participants (3%) failed either or both attention check questions, and one additional participant self-reported completing some rating scales in the reverse, so they also failed the attention check (4%).

**Table 1.** Participant and Study Characteristics

	Sample
Participant characteristics	
Age in years: *M* (SD)	15.2 (1.8)
Gender: *N* (%)	
Male	30 (25.0)
Female	89 (74.2)
Other	1 (0.8)
Socioeconomic status: *N* (%)	
Category 1 (low)	16 (13.5)
Category 2 Category 3	37 (31.1) 10 (8.4)
Category 4	9 (7.6)
Category 5 (high)	47 (39.5)
Participant has tried smoking: *N* (%)	
Yes	20 (16.7)
No	100 (83.3)
At least one family member smokes: *N* (%)	
Yes	30 (25.0)
No	83 (69.2)
Do not know	7 (5.8)
At least one friend smokes: *N* (%)	
Yes	49 (40.8)
No	55 (45.8)
Do not know	16 (13.3)
Participant has tried vaping: *N* (%)	
Yes	28 (23.3)
No	92 (76.7)
At least one family member vapes: *N* (%)	
Yes	29 (24.2)
No	86 (71.7)
Do not know	5 (4.2)
At least one friend vapes: *N* (%)	
Yes	59 (49.2)
No	49 (40.8)
Do not know	12 (10.0)
Smoking susceptibility: *M* (SD)	4.1 (1.7)
Vaping susceptibility: *M* (SD)	4.7 (1.9)
Study characteristics	
Study duration: *N* (%)	
Completed in 30 minutes or less	89 (74.2)
Did not complete in 30 minutes or less	31 (25.8)
Failed the attention checks: *N* (%)	
Yes	5 (4.2)
No	115 (95.8)

*N* = 120 for all variables except “socioeconomic status” (*N* = 119), as one participant reported not going to school. Therefore, school postcode was missing, and socioeconomic status could not be estimated. “Smoking susceptibility” and “vaping susceptibility” scores had a possible range from 3 (low) to 12 (high). The estimated survey response time was 30 minutes. “Failed the attention checks” included participants who answered either or both attention questions incorrectly and one additional participant who self-reported accidentally completing some rating scales in the reverse. *M* = mean, SD = standard deviation.

### Primary Analyses (Flavored Vs. Unflavored)

There was evidence of an effect of e-liquid packaging flavor description on packaging appraisal (mean difference 5.9, 95% CI: 4.2 to 7.6, *p* < .001, Cohen’s *dz* = 0.6; flavored: *M* = 33.3, SD = 16.2; unflavored: *M* = 27.3, SD* *= 14.6), and packaging receptivity (mean difference 4.2, 95% CI: 2.8 to 5.6, *p* < .001, Cohen’s *dz* = 0.5; flavored: *M* = 25.1, SD = 17.7; unflavored: *M* = 20.9, SD = 15.2; Table 2). There was evidence of an effect of e-liquid packaging flavor description on perceived audience (mean difference −5.2, 95% CI: −7.3 to −3.1, *p* < .001, Cohen’s *dz *= 0.4), where e-liquids with flavored descriptions were perceived as less grown-up (*M* = 61.9, SD* *= 18.8) than e-liquids with unflavored descriptions (*M *= 67.1, SD = 19.2). There was no clear evidence of a difference in perceived harm (mean difference −1.0, 95% CI: −2.6 to 0.5, *p* = .189, Cohen’s *dz* = 0.1; flavored: *M* = 66.8, SD = 19.7; unflavored: *M* = 67.9, SD = 19.3).

**Table 2.** Effect of E-liquid Packaging Flavor Description on Each Outcome

	Packaging appraisal			Packaging receptivity			Perceived harm			Perceived audience		
	*M* (95% CI)	*t*	*p*	*M* (95% CI)	*t*	*p*	*M* (95% CI)	*t*	*p*	*M* (95% CI)	*t*	*p*
*Primary analyses*												
Flavored	33.3 (30.3, 36.2)	—	—	25.1 (21.9, 28.3)	—	—	66.8 (63.3, 70.4)	—	—	61.9 (58.5, 65.3)	—	—
Unflavored	27.3 (24.7, 30.0)	—	—	20.9 (18.2, 23.7)	—	—	67.9 (64.4, 71.3)	—	—	67.1 (63.6, 70.6)	—	—
Difference	5.9 (4.2, 7.6)	6.9	<.001	4.2 (2.8, 5.6)	5.9	<.001	−1.0 (−2.6, 0.5)	−1.3	.189	−5.2 (−7.3, −3.1)	−4.9	<.001
*Secondary analyses*												
Sweet flavor	33.5 (30.4, 36.5)	—	—	25.3 (22.0, 28.6)	—	—	66.9 (63.2, 70.6)	—	—	60.4 (57.0, 63.9)	—	—
Fruit flavor	33.1 (30.1, 36.0)	—	—	24.8 (21.6, 28.1)	—	—	66.8 (63.1, 70.4)	—	—	63.4 (59.8, 66.9)	—	—
Difference	0.4 (−1.0, 1.7)	0.6	.566	0.4 (−0.7, 1.6)	0.7	.465	0.1 (−1.8, 2.0)	0.1	.921	−3.0 (−4.7, −1.2)	−3.3	.001

*N* = 120. Paired sample *t*-tests. *M* = mean, 95% CI = 95% confidence interval. For primary analyses, “difference” = flavored minus unflavored. For secondary analyses, “difference” = sweet flavor minus fruit flavor. “Packaging appraisal” and “packaging receptivity” scores had possible ranges from 0 (low) to 100 (high). “Perceived harm” scores had a possible range from 0 (not at all harmful) to 100 (very harmful). “Perceived audience” scores had a possible range from 0 (childish) to 100 (grown-up).

### Secondary Analyses (Sweet Flavor Vs. Fruit Flavor)

There was no clear evidence of an effect of e-liquid packaging flavor description on packaging appraisal (mean difference 0.4, 95% CI: −1.0 to 1.7, *p* = .566, Cohen’s *dz* = 0.1; sweet flavor: *M* = 33.5, SD = 16.7; fruit flavor: *M* = 33.1, SD* *= 16.5), packaging receptivity (mean difference 0.4, 95% CI: −0.7 to 1.6, *p* = .465, Cohen’s *dz* = 0.1; sweet flavor: *M* = 25.3, SD = 18.0; fruit flavor: *M* = 24.9, SD* *= 17.9), or perceived harm (mean difference 0.1, 95% CI: −1.8 to 2.0, *p* = .921, Cohen’s *dz* = 0.01; sweet flavor: *M *= 66.9, SD = 20.5; fruit flavor: *M* = 66.8, SD = 20.3; Table 2). However, there was evidence of an effect on perceived audience (mean difference −3.0, 95% CI: −4.7 to −1.2, *p* = .001, Cohen’s *dz* = 0.3), where e-liquids with sweet flavor descriptions were perceived as less grown-up (*M *= 60.4, SD = 19.1) than e-liquids with fruit flavor descriptions (*M* = 63.4, SD = 19.7).

### Sensitivity Analyses

Results did not qualitatively differ in the sample that excluded participants who failed the attention checks (*N* = 115; Table S2) compared to the full sample (*N* = 120).

### Unplanned Post hoc Analyses

Participants’ perceptions of “childish” to “grown-up” were explored by correlating this measure with the “not meant for someone like me” to “meant for someone like me” measure. There was a weak negative association (flavored: *r* = −0.2, *p* = .013; unflavored: *r* = −0.2, *p* = .047) indicating that e-liquids perceived as “grown-up” tended to be perceived as “not meant for someone like me.” In addition, the free-text comments were grouped into themes (Table 3) to help evaluate the findings and provide avenues for future research. Themes included other types of e-cigarette products, other aspects of packaging, other aspects of e-cigarettes, misunderstanding of survey item 4 “not at all harmful” to “very harmful,” regulations and policies, promotion of e-cigarettes, and reasons for e-cigarette use.

**Table 3.** Free-Text Comments Left by Participants Grouped by Themes

Quote theme	Quote
Other types of e-cigarette products	
	*Show Elf Bars, not vape liquid—they are the appealing thing for teens not the refillable ones.*
	*I think that Elf Bars are also an attractive way of presenting vaping to young people.*
Other aspects of packaging	
	*It was interesting for me to see how I was attracted to the color of the packaging and the flavors, other than that they looked like medicine that would not interest me or seem eye-catching.*
	*Vapes feel marketed to people my age much more than the people they were made for. The bright colors and nice flavors tempt kids in.*
	*There should be way more diverse packaging, it was all basically the same and the repeats made it quite boring to go through. Plus, they were all pretty unattractive.*
	*It is hard to say whether the packaging would tempt me to smoke/vape because I am very unlikely to anyway, so my previous knowledge would probably inform my decision more than the packaging. Even so, the packets where you can’t see inside and that have bright colors seem less harmful than the ones where you can see the liquid and the ones that are plain.*
Other aspects of e-cigarettes	
	*Should include questions on if the smell from other people’s vapes tempts you to try it.*
Misunderstanding of survey item 4 “not at all harmful/ very harmful”	
	*I wasn’t really sure what the slider with the words “it is harmful” was asking me.*
	*Didn’t understand the harmful question.*
Regulations and policies	
	*People shouldn’t be selling vapes to underage people in the first place because it’s illegal and the dangers to their health are massively increased if vaping/smoking is started young. It shouldn’t be so easily available, and people need to be educated more on the risks involved.*
	*I know that even in year 8 of my high school many people have vapes and it carries on through the years and how when I walk into the toilet there is always some sort of vape cloud in there smelling like apples or bubble gum etc. I also know how colorful and bright the e-liquids are in a store visible on display, not to mention the amount of vape stores there are around without much security to getting a vape (probably how so many people at school have them). Personally, I don’t like the idea fully but there hasn’t been much I can do about it.*
	*I believe vaping has become a growing issue because of how easily accessible it is to young people as well as it being affordable.*
Promotion of e-cigarettes	
	*Vaping has become so widespread with not only teenagers nearly 18 but also much younger even year 7. The marketing vape companies use is so clearly targeted towards a younger age demographic, so children get addicted to nicotine at a young age.*
	*TikTok plays a huge influence when young people see celebrities use it.*
Reasons for e-cigarette use	
	*There is a lot of influence of teenagers on their peers and younger kids to vape these days. Smoking seems to have decreased in high schools, only my friends who are at college or dropped out smoke, hardly anyone at sixth form unless it’s a party- but even then, far more people vape. It’s upsetting how many do. I have tried a vape and I’m very ashamed of it. But I didn’t gain satisfaction from it. I really think pressure to be “cool” and keeping up with trends is what is influencing vaping among younger people, and then people get addicted to the nicotine and enjoy the flavor.*
	*I also know parents who encourage or vape with their children, it has really become a culture/norm. I see my friends vape when they’re stressed or doing seemingly “cool” breaths with the vape to impress. My younger siblings (ages 13 and 14) find that friends receive money from their parents to buy vapes, but their parents don’t know. Vaping to cope with mental health even though young people know how bad it is. Young people also sell to other young people. I personally don’t vape because I have comfort at home with food and a home, know the health risks, and don’t want to buy into the herd mentality.*

## Discussion

In support of hypotheses, packaging appraisal and receptivity ratings were higher (more positive) for e-liquids with flavored versus unflavored descriptions. However, packaging appraisal and receptivity ratings were similar for e-liquids with sweet flavor and fruit flavor descriptions. As predicted, adolescents perceived e-liquids with flavored (vs. unflavored) and sweet flavor (vs. fruit flavor) descriptions as less grown-up. We did not find evidence for an effect of e-liquid packaging flavor description on perceived harm. This study indicates that flavor descriptions are one aspect of e-liquid packaging that influences e-cigarette product perceptions among nonsmoking and non-vaping adolescents, and it supports previous studies which suggest that flavored e-cigarette products have greater appeal to youth than unflavored products.^25,26^ By comparing flavored versus unflavored descriptions, this research also adds to previous literature that has compared subjective ratings of tobacco flavor versus non-tobacco flavor descriptions.^27,28,37^

Despite observing differences between the categories of flavor descriptions, overall, adolescents had low appraisal and receptivity for e-liquids, and they perceived them as being grown-up and harmful. Many have argued for the removal of flavored e-cigarette products from the market to protect youth.^11,38^ However, any policy changes that aim to prevent youth who do not smoke from vaping must be balanced, given the possible unintended consequences of policy changes and potential benefits of e-cigarettes and their flavors for some adult smokers.^30,31^ For example, among 18–25-year-olds in the United States, higher tax rates on electronic nicotine delivery systems are associated with decreased use of these products, but increased use of cigarettes.^39^ Some evidence suggests that banning all e-liquids except unflavored, tobacco-flavored, and menthol-flavored e-liquids would negatively impact some adult smokers and vapers by leading to continued smoking or relapses to smoking.^32^ However, many adult smokers have not tried e-cigarettes, reporting concerns about addiction, safety, and ineffectiveness, and others have tried and stopped using e-cigarettes, reporting insufficient relief of nicotine cravings, feeling unwell, and not liking the taste.^40^ Furthermore, one meta-analysis found e-cigarette use was associated with increased smoking cessation in randomized clinical trials but not in observational studies.^41^

The study has limitations. First, the analyses which compared sweet and fruit flavor descriptions were underpowered. Future studies with larger samples are needed to examine this distinction. Second, only five examples of flavor descriptions for each category were used. Therefore, the findings may not apply to other sweet and fruit flavors. Third, the framing of the question “can you tell me the number that best describes the packaging” may have been unsuitable for the “perceived harm” item. Two participants commented that they did not understand this question, and if this confusion was more pervasive, it may have contributed to the null findings for this outcome. Referencing “product” instead of “packaging” and specifying health harms may enhance clarity in a future study. Fourth, attrition was high (29%) and some participants took longer than predicted to complete the survey (26%; eg, they may have taken breaks), which could have led to bias. The number of questions may have been burdensome for adolescents and this was not pilot tested. Finally, we did not include an internal validity test such as a “stability test,” which involves repeating the same image and questions twice to check for consistent responses, as this would have made the survey longer.

Further research is needed to identify differences in flavor appeal between youth and adult smokers. For example, if certain e-liquid flavors have high appeal for youth regardless of the level of appeal for adult smokers, this could lead to an increase in harm if youth become addicted to nicotine.^27^ Whereas, if certain e-liquid flavors have high appeal for adult smokers, but low appeal for youth, the availability of these products could lead to harm reduction if their use causes adult smokers to completely substitute cigarettes for e-cigarettes.^27^ Future research could also use disposable vape stimuli to examine whether findings replicate across different e-cigarette devices. The popularity of different devices among 11–17-year-olds in Great Britain has changed significantly in the 3 years since designing this study. In 2022, the most frequently used e-cigarette product by 11–17-year-olds was a disposable vape (52% vs. 7% in 2020), with Elf Bar and Geek Bar being the most popular brands.^5^ In contrast, use of rechargeable tank-style e-cigarettes which can be filled with e-liquid has dropped (20% vs. 46% in 2020).

This study examined one aspect of e-liquid packaging (flavor descriptions) and we purposely selected brands with consistent packaging designs to isolate this effect. However, multiple factors such as colors, imagery (eg, cartoon characters), branding, and advertising—which often vary across flavors—could influence youth appeal.^11,42^ This point was echoed by some participants. Some researchers have argued that banning cartoons and brand imagery, for example, may reduce youth appeal without compromising the appeal of e-cigarettes to adult smokers.^5,22^ Indeed, one recent study found that standardizing e-cigarette packaging by removing brand imagery was associated with reduced e-cigarette appeal among youth but not among adults.^43^ According to one UK study, adult vapers acknowledge that protecting youth from the harms of e-cigarettes is an important regulatory requirement, but they believe that this should be achieved via childproofing, age limits, advertising restrictions, and health warnings, rather than the restriction of flavors.^44^ In January 2024, the UK Government announced that disposable vapes would be banned, and there would be new powers to restrict e-cigarette flavors, introduce plain packaging, and change how e-cigarettes are displayed in shops to reduce youth appeal.^24^

## Conclusions

Compared to e-liquids with unflavored descriptions, adolescents who do not smoke or vape appraised e-liquids with flavored descriptions more positively, were more receptive to using them, and perceived them to be less grown-up. However, despite these differences, adolescents had low appraisal and receptivity for e-liquids generally, and they perceived them as being grown-up and harmful. Further research is needed to examine adolescents’ ratings of flavor descriptions on alternative e-cigarette products and other aspects of e-cigarette packaging and promotion. Determining the factors driving the rise in youth vaping is vital to protect this vulnerable population. However, any policy changes must avoid compromising the appeal of e-cigarettes as a harm-reduction tool for adult smokers.

## Supplementary Material

ntae054_suppl_Supplementary_Material

## Data Availability

Study data, analysis code, and associated documents are publicly available at the University of Bristol data repository (doi:10.5523/bris.qmqm15cey1ef27u3szoujxt5x).

## References

[1] Kim J , Lee S, Chun J. An international systematic review of prevalence, risk, and protective factors associated with young people’s e-cigarette use. Int J Environ Res Public Health. 2022;19(18):11570. 36141845 10.3390/ijerph191811570 PMC9517489

[2] Becker TD , Rice TR. Youth vaping: a review and update on global epidemiology, physical and behavioral health risks, and clinical considerations. Eur J Pediatr. 2022;181(2):453–462. 34396473 10.1007/s00431-021-04220-x PMC8364775

[3] Chan GCK , Gartner C, Lim C, et al. Association between the implementation of tobacco control policies and adolescent vaping in 44 lower-middle, upper-middle, and high-income countries. Addiction. 2022;117(8):2296–2305. 35545233 10.1111/add.15892

[4] NHS Digital. Smoking, Drinking and Drug Use among Young People in England, 2021. 2022. https://digital.nhs.uk/data-and-information/publications/statistical/smoking-drinking-and-drug-use-among-young-people-in-england/2021/part-4-electronic-cigarette-use-vaping

[5] Action on Smoking and Health. Use of e-cigarettes (vapes) among young people in Great Britain. 2022.

[6] Action on Smoking and Health. Use of e-cigarettes (vapes) among young people in Great Britain. 2023. https://ash.org.uk/resources/view/use-of-e-cigarettes-among-young-people-in-great-britain

[7] Hartmann-Boyce J , McRobbie H, Lindson N, et al. Electronic cigarettes for smoking cessation. Cochrane Database Syst Rev. 2021;4:CD010216. 33913154 10.1002/14651858.CD010216.pub5 PMC8092424

[8] Benowitz NL. Nicotine addiction. N Engl J Med. 2010;362(24):2295–2303. 20554984 10.1056/NEJMra0809890 PMC2928221

[9] Bush A , Bhatt JM, Connett GJ, et al. A public health emergency among young people. Lancet Respir Med. 2020;8(3):231–233. 31843487 10.1016/S2213-2600(19)30468-0

[10] Soneji S , Barrington-Trimis JL, Wills TA, et al. Association between initial use of e-cigarettes and subsequent cigarette smoking among adolescents and young adults: a systematic review and meta-analysis. JAMA Pediatr. 2017;171(8):788–797. 28654986 10.1001/jamapediatrics.2017.1488 PMC5656237

[11] Ferkol TW , Farber HJ, La Grutta S, et al; Forum of International Respiratory Societies. Electronic cigarette use in youths: a position statement of the Forum of International Respiratory Societies. Eur Respir J. 2018;51(5):1800278. 29848575 10.1183/13993003.00278-2018

[12] Singh S , Windle SB, Filion KB, et al. E-cigarettes and youth: patterns of use, potential harms, and recommendations. Prev Med. 2020;133:106009. 32027913 10.1016/j.ypmed.2020.106009

[13] Chan GCK , Stjepanovic D, Lim C, et al. Gateway or common liability? A systematic review and meta-analysis of studies of adolescent e-cigarette use and future smoking initiation. Addiction. 2021;116(4):743–756. 32888234 10.1111/add.15246

[14] Khouja JN , Suddell SF, Peters SE, Taylor AE, Munafo MR. Is e-cigarette use in non-smoking young adults associated with later smoking? A systematic review and meta-analysis. Tob Control. 2020;30:8–15. 32156694 10.1136/tobaccocontrol-2019-055433 PMC7803902

[15] Khouja JN , Wootton RE, Taylor AE, Davey Smith G, Munafo MR. Association of genetic liability to smoking initiation with e-cigarette use in young adults: a cohort study. PLoS Med. 2021;18(3):e1003555. 33735204 10.1371/journal.pmed.1003555 PMC7971530

[16] World Health Organisation. *WHO report on the global tobacco epidemic 2021: addressing new and emerging products*. 2021. https://www.who.int/publications/i/item/9789240032095

[17] Krusemann EJZ , Boesveldt S, de Graaf K, Talhout R. An e-liquid flavor wheel: a shared vocabulary based on systematically reviewing e-liquid flavor classifications in literature. Nicotine Tob Res. 2019;21(10):1310–1319. 29788484 10.1093/ntr/nty101 PMC6751518

[18] US Food and Drug Administration. Statement from FDA Commissioner Scott Gottlieb, M.D., on new enforcement actions and a Youth Tobacco Prevention Plan to stop youth use of, and access to, JUUL and other e-cigarettes. 2018. https://www.fda.gov/news-events/press-announcements/statement-fda-commissioner-scott-gottlieb-md-new-enforcement-actions-and-youth-tobacco-prevention

[19] Notley C , Gentry S, Cox S, et al. Youth use of e-liquid flavours-a systematic review exploring patterns of use of e-liquid flavours and associations with continued vaping, tobacco smoking uptake or cessation. Addiction. 2022;117(5):1258–1272. 34784651 10.1111/add.15723 PMC9299186

[20] Meernik C , Baker HM, Kowitt SD, Ranney LM, Goldstein AO. Impact of non-menthol flavours in e-cigarettes on perceptions and use: an updated systematic review. BMJ Open. 2019;9(10):e031598. 10.1136/bmjopen-2019-031598 PMC6797351 31619431

[21] Zhang H , Wang Y, Shen L, Gu Y, Shao F. E-cigarette use and regulation: a comparative analysis between the United States, the UK, and China. Am J Bioeth. 2022;22(10):29–31. 10.1080/15265161.2022.2110971 36170090

[22] Khan J. *The Khan review: making smoking obsolete* . 2022. https://www.gov.uk/government/publications/the-khan-review-making-smoking-obsolete

[23] MHRA. E-cigarettes: regulations for consumer products. 2022. https://www.gov.uk/guidance/e-cigarettes-regulations-for-consumer-products

[24] UK Government. *Disposable vapes banned to protect children’s health*. 2024. https://www.gov.uk/government/news/disposable-vapes-banned-to-protect-childrens-health

[25] Vasiljevic M , Petrescu DC, Marteau TM. Impact of advertisements promoting candy-like flavoured e-cigarettes on appeal of tobacco smoking among children: an experimental study. Tob Control. 2016;25(e2):e107–e112. 26781305 10.1136/tobaccocontrol-2015-052593 PMC5284337

[26] Cooper M , Harrell MB, Perez A, Delk J, Perry CL. Flavorings and perceived harm and addictiveness of e-cigarettes among youth. Tob Regul Sci. 2016;2(3):278–289. 27722185 10.18001/TRS.2.3.7 PMC5049876

[27] Pepper JK , Ribisl KM, Brewer NT. Adolescents’ interest in trying flavoured e-cigarettes. Tob Control. 2016;25(suppl 2):iiii62–iiii66. 10.1136/tobaccocontrol-2016-053174 PMC5125087 27633762

[28] Ford A , MacKintosh AM, Bauld L, Moodie C, Hastings G. Adolescents’ responses to the promotion and flavouring of e-cigarettes. Int J Public Health. 2016;61(2):215–224. 26650455 10.1007/s00038-015-0769-5 PMC4819499

[29] McKelvey K , Baiocchi M, Ramamurthi D, McLaughlin S, Halpern-Felsher B. Youth say ads for flavored e-liquids are for them. Addict Behav. 2019;91:164–170. 30314868 10.1016/j.addbeh.2018.08.029 PMC6663555

[30] McNeill A , Brose LS, Calder R, Bauld L, Robson D. *Vaping in England: an evidence update including mental health and pregnancy, March 2020: a report commissioned by Public Health England* . 2020. https://www.gov.uk/government/publications/vaping-in-england-evidence-update-march-2020 10.1093/ntr/ntab017 PMC8372638 33538828

[31] Department of Health & Social Care. Creating a smokefree generation and tackling youth vaping: your views. 2023. https://www.gov.uk/government/consultations/creating-a-smokefree-generation-and-tackling-youth-vaping/creating-a-smokefree-generation-and-tackling-youth-vaping-your-views

[32] Khouja JN , Dyer ML, Havill MA, Dockrell MJ, Munafò MR, Attwood AS. Exploring the opinions and potential impact of unflavoured e-liquid on smoking cessation among UK smokers and smoking relapse among UK e-cigarette users: Findings from a qualitative study. 2022. https://www.researchsquare.com/article/rs-2054093/v1

[33] Duyff RL , Birch LL, Byrd-Bredbenner C, et al. Candy consumption patterns, effects on health, and behavioral strategies to promote moderation: summary report of a roundtable discussion. Adv Nutr. 2015;6(1):139S–146S. 25593156 10.3945/an.114.007302 PMC4288276

[34] Watts C , Egger S, Dessaix A, et al. Vaping product access and use among 14-17-year-olds in New South Wales: a cross-sectional study. Aust N Z J Public Health. 2022;46(6):814–820. 36156328 10.1111/1753-6405.13316

[35] Office for Students. About POLAR and adult HE: Young participation by area. https://www.officeforstudents.org.uk/data-and-analysis/young-participation-by-area/about-polar-and-adult-he/

[36] Ford A , Mackintosh AM, Moodie C, Richardson S, Hastings G. Cigarette pack design and adolescent smoking susceptibility: a cross-sectional survey. BMJ Open. 2013;3(9):e003282. 10.1136/bmjopen-2013-003282 PMC3780301 24056481

[37] Strombotne K , Buckell J, Sindelar JL. Do JUUL and e-cigarette flavours change risk perceptions of adolescents? Evidence from a national survey. Tob Control. 2021;30(2):199–205. 32300029 10.1136/tobaccocontrol-2019-055394 PMC7572758

[38] Drazen JM , Morrissey S, Campion EW. The dangerous flavors of e-cigarettes. N Engl J Med. 2019;380(7):679–680. 30699053 10.1056/NEJMe1900484

[39] Friedman AS , Pesko MF. Young adult responses to taxes on cigarettes and electronic nicotine delivery systems. Addiction. 2022;117(12):3121–3128. 35852452 10.1111/add.16002 PMC9796020

[40] Action on Smoking and Health. Use of e-cigarettes (vapes) among adults in Great Britain. 2023. https://ash.org.uk/uploads/Use-of-e-cigarettes-among-adults-in-Great-Britain-2023.pdf

[41] Wang RJ , Bhadriraju S, Glantz SA. E-cigarette use and adult cigarette smoking cessation: A meta-analysis. Am J Public Health. 2021;111(2):230–246. 33351653 10.2105/AJPH.2020.305999 PMC7811087

[42] Hardie L , McCool J, Freeman B. Online retail promotion of e-cigarettes in New Zealand: a content analysis of e-cigarette retailers in a regulatory void. Health Promot J Austr. 2022;33(1):91–98. 33565666 10.1002/hpja.464

[43] Taylor E , Arnott D, Cheeseman H, et al. Association of fully branded and standardized e-cigarette packaging with interest in trying products among youths and adults in Great Britain. JAMA Netw Open. 2023;6(3):e231799. 36917111 10.1001/jamanetworkopen.2023.1799 PMC10015302

[44] Farrimond H. E-cigarette regulation and policy: UK vapers’. Addiction. 2016;111(6):1077–1083. 26802864 10.1111/add.13322

