# Supplementary material for: Do Flavor Descriptions Influence Subjective Ratings of Flavored and Unflavored E-liquids Among Nonsmoking and Non-vaping UK Adolescents?
Source: Nicotine Tob Res. 2024 Mar 12;26(9):1141–9. doi: 10.1093/ntr/ntae054 (PMC11339169; doi:10.1093/ntr/ntae054)
Supplement: ntae054_suppl_Supplementary_Material [file ntae054_suppl_supplementary_material.pdf]

Do Flavour Descriptions Influence Subjective Ratings of Flavoured and Unflavoured E-Liquids among Non-Smoking and Non-Vaping UK Adolescents?

Maddy L. Dyer, PhD[1,2], Steph F. Suddell, PhD[3], Jasmine N. Khouja, PhD[1,2], Michelle A. Havill, MScMed[4], Anna K. M. Blackwell, PhD[5], Olivia M. Maynard, PhD[1,2], Marcus R. Munafò, PhD[1,2,6], and Angela S. Attwood, PhD[1,2]

[1] School of Psychological Science, University of Bristol, Bristol, UK.

[2] Medical Research Council Integrative Epidemiology Unit at the University of Bristol, Bristol, UK.

[3] Trinity College Institute of Neuroscience (TCIN), Trinity College Dublin, Dublin, Ireland.

[4] Office for Health Improvement and Disparities, Department of Health and Social Care, London, UK.

[5] Department of Psychology, University of Bath, Bath, UK.

[6] National Institute for Health Research Bristol Biomedical Research Centre, University Hospitals Bristol NHS Foundation Trust, Bristol, UK.

Corresponding author: Jasmine N. Khouja, School of Psychological Science, University of Bristol, 12a Priory Road, Bristol, BS8 1TU, UK. Email: jasmine.khouja@bristol.ac.uk. ORCID: https://orcid.org/0000-0002-7944-2981

Supplementary Table S1. Flavour descriptions for the 15 e-liquid stimuli

<table>
<tr><th>Fruit flavour</th><th>Sweet flavour</th><th>Unflavoured</th></tr>
<tr><td>Watermelon
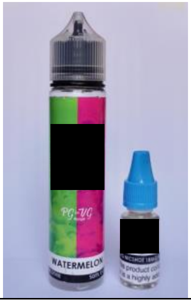</td><td>Tutti Frutti
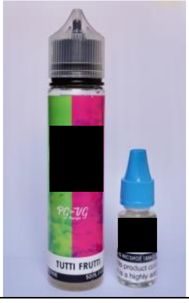</td><td>Unflavoured
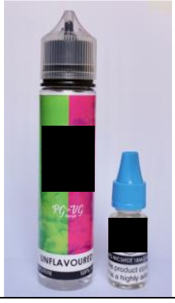</td></tr>
<tr><td>Strawberry
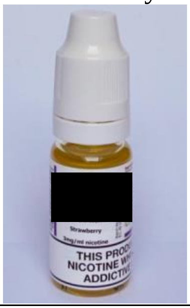</td><td>Pear Drops
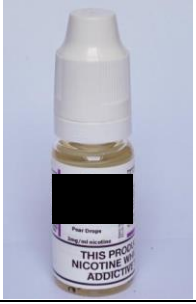</td><td>Flavourless
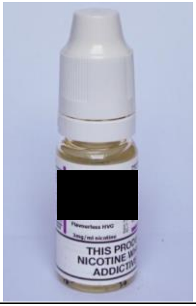</td></tr>
<tr><td>Blueberry
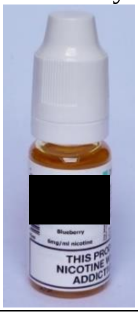</td><td>Bubble Gum
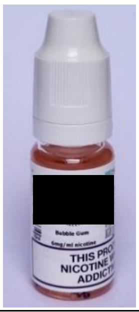</td><td>Flavourless
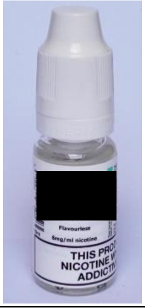</td></tr>
<tr><td>Blackcurrant
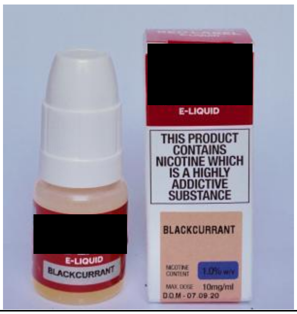</td><td>Bubble Gum
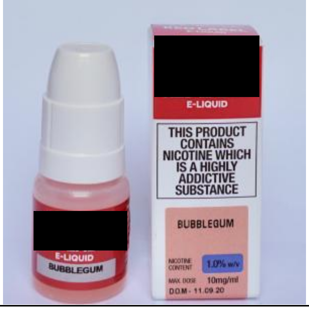</td><td>Unflavoured
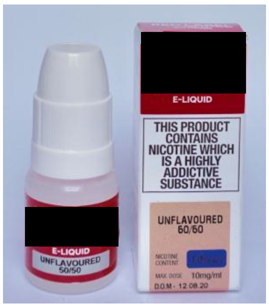</td></tr>
<tr><td>Peach Swirl
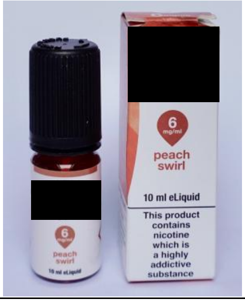</td><td>Caramel Marshmallow
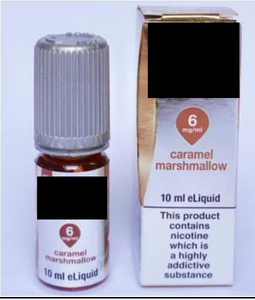</td><td>Pure
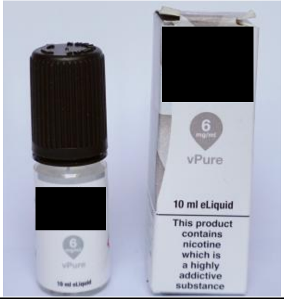</td></tr>
</table>

Photographs were taken of each product under standardised conditions. When both a bottle and a box were supplied, the bottle was photographed adjacent to its box. For one brand, the largest volume of e-liquid available to purchase was 50ml. The nicotine was supplied separately in the form of a nicotine shot due to UK regulation that restricts the maximum volume of nicotine-containing e-liquid for sale in one refill container to 10ml (https://www.gov.uk/guidance/e-cigarettes-regulations-for-consumer-products). In this case, the bottle of e-liquid was photographed adjacent to the nicotine shot. Images presented here are smaller than those presented on screen during the study. Brand names have been removed.

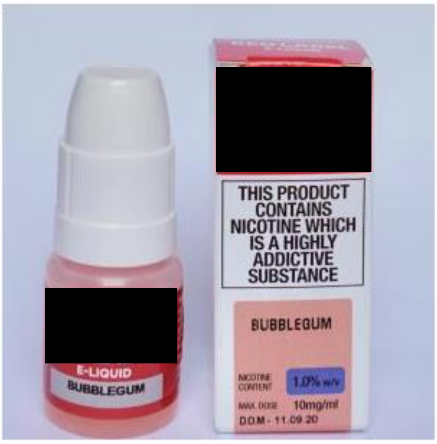

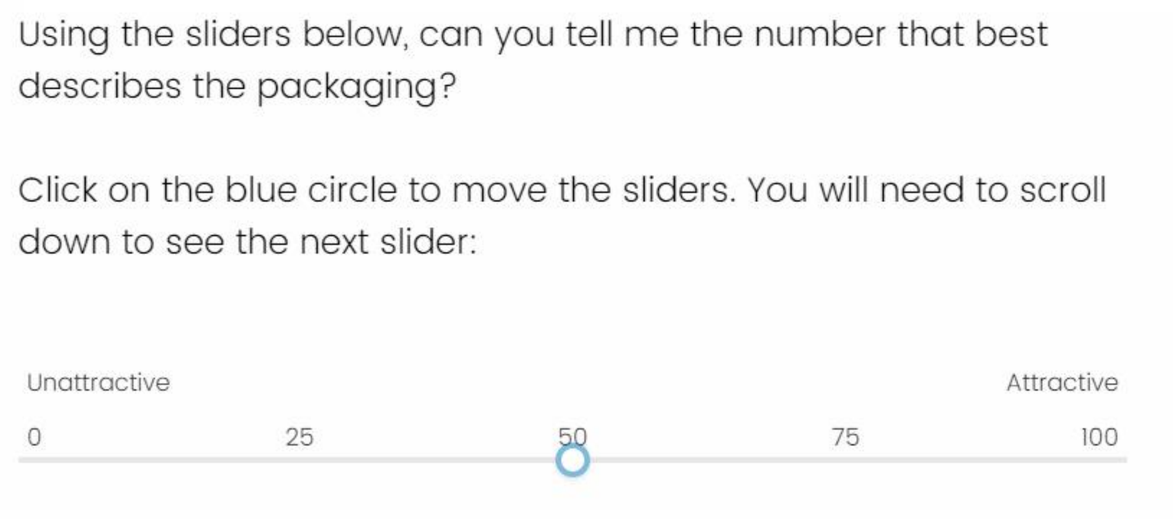

Supplementary Figure S1. Example image and rating scale presentation.

Brand names have been removed.

Supplementary Table S2. Effect of e-liquid packaging flavour description on each outcome, excluding participants who failed the attention checks

	**Packaging appraisal**			**Packaging receptivity**			**Perceived harm**			**Perceived audience**		
	**M (95% CI)**	***t***	***p***	**M (95% CI)**	***t***	***p***	**M (95% CI)**	***t***	***p***	**M (95% CI)**	***t***	***p***
**Primary analyses**												
Flavoured	33.3 (30.5, 36.2)	-	-	25.1 (22.0, 28.3)	-	-	67.4 (63.9, 70.9)	-	-	63.0 (59.8, 66.2)	-	-
Unflavoured	27.2 (24.6, 29.7)	-	-	20.8 (18.2, 23.5)	-	-	68.2 (64.8, 71.6)	-	-	68.6 (65.4, 71.8)	-	-
Difference	6.2 (4.4, 8.0)	6.9	<.001	4.3 (2.9, 5.7)	5.9	<.001	-0.8 (-2.4, 0.8)	-1.0	.305	-5.6 (-7.8, -3.4)	-5.1	<.001
**Secondary analyses**												
Sweet flavour	33.6 (30.6, 36.5)	-	-	25.4 (22.2, 28.6)	-	-	67.5 (63.8, 71.1)	-	-	61.5 (58.2, 64.8)	-	-
Fruit flavour	33.1 (30.2, 36.1)	-	-	24.9 (21.7, 28.0)	-	-	67.3 (63.6, 70.9)	-	-	64.5 (61.1, 67.8)	-	-
Difference	0.4 (-0.9, 1.8)	0.6	.526	0.5 (-0.7, 1.8)	0.9	.373	0.2 (-1.8, 2.2)	0.2	.832	-3.0 (-4.8, -1.2)	-3.2	.002

N = 115. Paired sample t-tests. M = mean, 95% CI = 95% confidence interval. For primary analyses, 'difference' = flavoured minus unflavoured. For secondary analyses, 'difference' = sweet flavour minus fruit flavour. 'Packaging appraisal' and 'packaging receptivity' scores had possible ranges from 0 (low) to 100 (high). 'Perceived harm' scores had a possible range from 0 (not at all harmful) to 100 (very harmful). 'Perceived audience' scores had a possible range from 0 (childish) to 100 (grown-up).
